# Distribution of clonal hematopoiesis of indeterminate potential (CHIP) is not associated with race in patients with plasma cell neoplasms

**DOI:** 10.1038/s41408-022-00706-5

**Published:** 2022-07-26

**Authors:** Marie-France Gagnon, Shulan Tian, Susan Geyer, Neeraj Sharma, Celine M. Vachon, Yael Kusne, P. Leif Bergsagel, A. Keith Stewart, S. Vincent Rajkumar, Shaji Kumar, Sikander Ailawadhi, Linda B. Baughn

**Affiliations:** 1 Division of Laboratory Genetics and Genomics, Department of Laboratory Medicine and Pathology, Mayo Clinic, Rochester, MN USA; 2 Division of Computational Biology, Department of Quantitative Health Sciences, Mayo Clinic, Rochester, MN USA; 3 Division of Clinical Trials and Biostatistics, Department of Quantitative Health Sciences, Mayo Clinic, Rochester, MN USA; 4 Division of Epidemiology, Department of Quantitative Health Sciences, Mayo Clinic, Rochester, MN USA; 5 Division of Hematology, Department of Internal Medicine, Mayo Clinic, Scottsdale, AZ USA; 6 Princess Margaret Cancer Centre, Toronto, ON Canada; 7 Division of Hematology, Department of Internal Medicine, Mayo Clinic, Rochester, MN USA; 8 Division of Hematology, Department of Internal Medicine, Mayo Clinic, Jacksonville, FL USA; 9 Division of Hematopathology, Department of Laboratory Medicine and Pathology, Mayo Clinic, Rochester, MN USA

**Keywords:** Cancer genomics, Myeloma

Dear Editor,

Several studies have recently raised mounting interest regarding clonal hematopoiesis (CH) in the setting of plasma cell neoplasms (PCNs). CH has been shown to occur at an increased frequency among patients with multiple myeloma (MM) undergoing autologous stem cell transplantation and to adversely affect overall survival (OS) and progression-free survival (PFS) in the absence of immunomodulatory drug maintenance [1]. While evidence regarding a role for CH in PCN disease biology is growing, research efforts have largely focused on patients who self-report as non-Hispanic White (NHW). Given the increased risk of MM among Black/AA individuals and the association between CHIP and MM progression, we sought to interrogate CH in a diverse cohort and compare the frequency of this condition in individuals who self-identify as Black/AA vs. NHW.

Following Mayo Clinic Institutional Review Board approval and patient informed consent, we performed targeted next-generation sequencing in a cohort of 174 patients with a PCN including MGUS, smoldering MM, MM, amyloidosis and other PCN. Samples were selected from Mayo Clinic patients with available DNA from the diagnostic bone marrow biopsy (subset from the cohort previously described in Baughn et al. [2]). Genomic DNA was extracted from bone marrow aspirates following a 24-h culture period using the QIAmp DNeasy Blood and Tissue Kit (Qiagen, Germantown, Maryland) and subjected to deep sequencing using a custom target bait panel including 30 genes recurrently mutated in CH. Libraries were sequenced on an Illumina HiSeq 4000 (average sequencing depth of ~4000x). Additional details regarding bioinformatics analyses and variant curation are available in Supplementary Materials. Allele frequency thresholds for CH were set at 0.01 and 0.02 as per the recognized definition of CHIP. Considering the overlap in genes mutated in CH and in MM, analyses were restricted to mutations in *DNMT3A, TET2* and *ASXL1* to ensure unambiguous attribution of mutations to the CH population. Patients were grouped according to self-reported race and ethnicity (Black/AA and NHW) and compared for CH frequency and outcome (OS and PFS). Survival and time-to-event curves were constructed using the Kaplan–Meier method and compared by the log-rank test. Cox proportional-hazards regression models were used for multivariable analysis to determine hazard ratios and associated confidence intervals. Detailed statistical methods and information regarding other self-reported racial groups are provided in Supplementary Materials.

The cohort included 174 patients with a PCN (91 (52%) cases of MM, 30 cases of MGUS (17%), 20 cases of smoldering MM (11%), 27 cases of amyloidosis (16%), 4 (2.3%) cases of POEMS, one case of Waldenstrom macroglobulinemia (0.6%) and one case of solitary plasmacytoma (0.6%) (Table 1). Median age was 65 years (range 34–89). Ninety-six patients (55%) were male and 105 (60%) received therapy for their PCN. Sixty-four (37%) self-identified as Black/AA and 81 (47%) as NHW. Self-reported race and ethnicity were highly concordant with calculated ancestry assessed through genotyping as previously demonstrated [2] (see Supplementary Results). Black/AA patients were younger than NHW patients (respective median age: 62 vs. 68 years, *p* value <0.001). The distribution of PCN types did not differ significantly between these two race/ethnicity groups. Translocations disrupting the *MAF or MAFB* oncogenes were more common among Black/AA vs. NHW patients (22% vs. 1%, *p* < 0.001). Groups did not differ regarding type of induction therapy and frequency of autologous stem cell transplantation (Table 1).

**Table 1:** Characteristics and baseline demographics of study cohort (*n* = 174).

Characteristic	All patients, *N* = 174^a^	Black/African American, *N* = 64^a^	Non-Hispanic White, *N* = 81^a^	*p* value^b^
Gender (male)	96 (55%)	34 (53%)	41 (51%)	0.8
Age	65 (56, 71)	62 (52, 66)	68 (59, 75)	<0.001
Diagnosis				
Multiple myeloma	91 (52%)	29 (45%)	45 (56%)	0.3
MGUS	30 (17%)	13 (20%)	12 (15%)	0.4
Smoldering multiple myeloma	20 (11%)	6 (9.4%)	13 (16%)	0.2
Amyloidosis	27 (16%)	13 (20%)	8 (9.9%)	0.1
POEMS	4 (2.3%)	3 (4.7%)	1 (1.2%)	0.3
Other	2 (1.1%)	0 (0%)	2 (2.5%)	0.5
Primary cytogenetic abnormality				
t(11;14)	45 (26%)	17 (27%)	16 (20%)	0.3
t(4;14)	9 (5.2%)	3 (4.7%)	5 (6.2%)	>0.9
t(6;14)	6 (3.4%)	1 (1.6%)	4 (4.9%)	0.4
MAF translocations	15 (8.6%)	14 (22%)	1 (1.2%)	<0.001
Trisomy no IGH	74 (43%)	23 (36%)	40 (49%)	0.1
Other IGH	17 (9.8%)	4 (6.2%)	10 (12%)	0.2
Bone marrow plasmacytosis	18 (5, 50)	15 (5, 40)	20 (10, 50)	0.2
Concurrent amyloidosis	34 (28%)	15 (43%)	10 (15%)	0.002
ISS at diagnosis				0.6
1	31 (42%)	14 (54%)	14 (42%)	
2	14 (19%)	4 (15%)	5 (15%)	
3	29 (39%)	8 (31%)	14 (42%)	
MSMART high risk category	28 (16%)	17 (27%)	9 (11%)	0.02
R-ISS at diagnosis				0.8
1	11 (24%)	6 (35%)	4 (25%)	
2	24 (53%)	8 (47%)	8 (50%)	
3	10 (22%)	3 (18%)	4 (25%)	
Paraprotein subtype				0.7
IgG	99 (58%)	39 (63%)	46 (57%)	
IgA	40 (24%)	14 (23%)	21 (26%)	
LCO	29 (17%)	9 (15%)	11 (14%)	
Other	2 (1.2%)	0 (0%)	2 (2.5%)	
Kappa light chain	98 (57%)	35 (55%)	45 (56%)	0.9
CH (AF threshold 0.01)	37 (21%)	9 (14%)	24 (30%)	0.026
*DNMT3A*	21 (12%)	7 (11%)	12 (15%)	0.5
*TET2*	21 (12%)	6 (9.4%)	13 (16%)	0.3
*ASXL1*	5 (2.9%)	2 (3.1%)	3 (3.7%)	>0.9
Maximal VAF	0.04 (0.02, 0.10)	0.02 (0.02, 0.04)	0.06 (0.02, 0.15)	0.2
VAF (additive)	0.08 (0.04, 0.24)	0.04 (0.03, 0.05)	0.27 (0.20, 0.48)	0.004
VAF (multiplicative)	0.00 (0.00, 0.01)	0.00 (0.00, 0.00)	0.01 (0.01, 0.01)	0.008
CH (AF threshold 0.02)	13 (7.5%)	3 (4.7%)	10 (12%)	0.10
*TET2*	8 (4.6%)	0 (0%)	8 (9.9%)	0.009
*DNMT3A*	4 (2.3%)	2 (3.1%)	2 (2.5%)	>0.9
*ASXL1*	2 (1.1%)	1 (1.6%)	1 (1.2%)	>0.9
Any treatment received	105 (60%)	40 (49%)	34 (53%)	0.7
Initial treatment regimen				
Proteasome inhibitor-based	39 (26%)	12 (22%)	18 (26%)	0.6
Immunomodulator-based	54 (36%)	20 (36%)	26 (38%)	0.9
Best response to initial treatment				0.06
Stringent complete response	2 (2.2%)	1 (3.1%)	0 (0%)	
Complete response	17 (18%)	4 (12%)	7 (18%)	
Very good partial response	31 (34%)	7 (22%)	17 (41%)	
Partial response	26 (28%)	15 (47%)	8 (20%)	
Minimal response	2 (2.2%)	1 (3.1%)	0 (0%)	
Stable disease	14 (15%)	4 (12%)	8 (20%)	
ASCT Received	44 (30%)	17 (32%)	16 (25%)	0.4
Progression of disease	64 (54%)	21 (47%)	28 (56%)	0.4
Death all cause	55 (32%)	21 (33%)	22 (27%)	>0.9
Death from PCN progression	12 (7%)	4 (6%)	6 (7%)	
Death from infection	5 (3%)	2 (3%)	2 (2%)	
Sudden death	2 (1%)	0 (0%)	0 (0%)	
Death from cardiovascular event	1 (1%)	0 (0%)	1 (1%)	
Death from other cause	5 (3%)	3 (5%)	2 (2%)	
Cause of death unknown	30 (17%)	12 (19%)	11 (14%)	

*ASCT* autologous stem cell transplantation, *LCO* light chain only, *MGUS* monoclonal gammopathy of undetermined significance, *VAF* variant allele frequency.

^a^Median (IQR); *n* (%).

^b^Displayed *p* values correspond to the comparison between Black/African American and non-Hispanic White individuals.

In our full cohort, CH (VAF ≥ 0.01) was detected in 21% (*n* = 37/174) of patients. Median allele frequency was 4% (range: 1–98.9%). CH was detected in 21 patients with MM (23% of 91), 5 with MGUS (17% of 30), 5 with SMM (25% of 20) and 6 with amyloidosis (22% of 27). When analyses were restricted to mutations with a VAF of ≥ 0.02, 13 mutations (7.5%) within *DNMT3A, TET2* and *ASXL1* were documented (4 with MM, 3 with MGUS, 2 with SMM, 4 with AL amyloidosis). When CH was classified based on VAF ≥ 0.01, patients with CH were significantly older than patients without CH (median age: 71 and 64 years, respectively, *p* value <0.001). When a VAF threshold of 0.02 was considered, no significant difference in age (median age: 66 vs. 65 years respectively, *p* = 0.26) was seen. CH, as defined by an VAF threshold ≥ 0.01, occurred at a lower frequency in Black/AA individuals (*n* = 9/64, 14%) as compared with NHW individuals (*n* = 24/81, 30%; *p* value = 0.03). In multivariable analysis, race and ethnicity were not significantly associated with the incidence of CH and age remained the significant predictor of CH frequency. These findings suggest that the lower incidence of CH in Black/AA patients was likely confounded by lower median age in our cohort of Black/AA patients. Among Black/AA individuals with CH, mutations in *DNMT3A* (*n* = 7, 11%) and *TET2* (*n* = 6, 9%) were most common. The individual frequencies of *DNMT3A, TET2* and *ASXL1* mutations did not significantly differ from those of NHW individuals when mutations with VAF ≥ 0.01 were considered. While the limited number of events calls for caution in the interpretation of data, *TET2* mutations appeared less prevalent in AA individuals when restricting analyses to mutations with allele frequencies of VAF ≥ 0.02 (0% vs. 9.9% respectively, *p* value = 0.009).

We next assessed and compared OS and PFS based on CH status and self-reported race and ethnicity. Given the differential definitions of progressive disease in various PCN types, PFS analyses were restricted to individuals diagnosed with MM (*n* = 74, 45 NHW, 29 Black/AA). No significant differences in PFS between Black/AA and NHW MM patients were observed (HR = 0.72, 95% CI: 0.41–1.28; *p* value = 0.26) (Fig. 1A). In the univariate setting, CH (VAF ≥ 0.01) tended to be associated with poorer PFS (HR = 1.43, 95% CI: 0.77–2.65; *p* value = 0.26). Although limited to only 4 (NHW) MM patients, CH with a VAF ≥ 0.02, was associated with a significantly worse PFS (HR = 5.52, 95% CI: 1.82–16.74; *p* value = 0.003) (Fig. 1B).

**Fig. 1:**
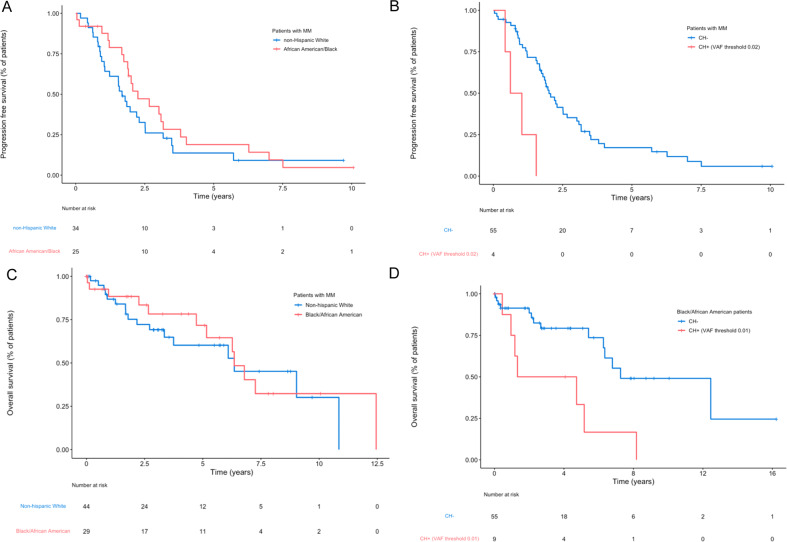
Progression-free survival and overall survival according to race, ethicity, PCN type and CH status. **A** Progression-free survival for patients with multiple myeloma according to race and ethnicity (HR = 0.72, 95% CI: 0.41–1.28; *p* = 0.26). **B** Progression-free survival for patients with multiple myeloma according to clonal hematopoiesis status (VAF ≥ 0.02) (HR = 5.52, 95% CI: 1.82–16.74; *p* = 0.003). **C** Overall survival of patients with multiple myeloma based on race and ethnicity. **D** Overall survival of Black/African American patients with PCN according to CH status (VAF ≥ 0.01) (HR = 4.57, 95% CI: 1.48–14.1; *p* = 0.008). CH clonal hematopoiesis, MM multiple myeloma, PCN plasma cell neoplasm.

In assessing OS of the patients who were NHW or Black/AA, the median follow-up was 46.9 months (95% CI: 34.8–64.4). OS was similar between Black/AA vs. NHW patients, even with stratification on PCN type (HR = 1.05, 95% CI: 0.53–1.86; *p* value = 0.99) (Fig. 1C, Supplementary Figs. 1 and 5). When evaluating the influence of CH (VAF ≥ 0.01), a tendency toward poorer OS for those with CH in comparison to those without (HR = 1.80, 95% CI: 0.24–3.53; *p* value = 0.088) was observed, even after adjustment for race group and stratification on PCN type. This association was more substantial when considering CH with a VAF ≥ 0.02 (HR = 3.93, 95% CI: 1.60–9.65; *p* value = 0.003). Adjusting for age in these models confounded these results, mostly due to the high multicollinearity between age and CH incidence. However, inclusion of CH status (VAF ≥ 0.02) yielded a better predictive model for OS than models with age. Among patients with available data, cause of death did not differ between patients with CH and without CH and was mostly related to PCN progression and infection (*p* value = 0.8). To explore potential effect modification based on race and ethnicity, we further evaluated OS within Black/AA and NHW patients. In the NHW patients, CH status using VAF ≥ 0.01 did not significantly influence OS when stratifying on PCN type (HR = 1.11, 95% CI: 0.43–2.82; *p* value = 0.82) (Supplementary Fig. 7). When applying the same model to Black/AA patients with PCN, CH was associated with significantly worse OS when stratifying on PCN type (HR = 4.57, 95% CI: 1.48–14.1; *p* value = 0.008) (Fig. 1D). The influence of CH was similar in NHW and Black/AA patients at a VAF threshold ≥ 0.02. Black/AA and NHW patient with MM with and without CH did not otherwise differ regarding additional prognostic factors of relevance suggesting that this effect was not attributable to differences in established prognostic features (Table 2).

**Table 2:** Characteristics of Black/AA and NHW patients with MM based on CH status.

	Black/AA without CH (*n* = 25)^a^	Black/AA with CH (*n* = 4)^a^	*p* value^b^	NHW without CH (*n* = 31)^a^	NHW with CH (*n* = 14)^a^	*p* value^b^
Age	65 (58, 68)	71 (70, 74)	0.013	67 (60, 76)	73 (67, 76)	0.2
Primary cytogenetics abnormality						
t(11;14)	3 (12%)	0 (0%)	>0.9	8 (26%)	3 (21%)	>0.9
t(4;14)	0 (0%)	0 (0%)		2 (6.5%)	1 (7.1%)	>0.9
t(6;14)	0 (0%)	0 (0%)		2 (6.5%)	1 (7.1%)	>0.9
MAF translocations	7 (28%)	2 (50%)	0.6	0 (0%)	0 (0%)	
Trisomy no IGH	13 (52%)	2 (50%)	>0.9	15 (48%)	5 (36%)	0.4
Other IGH	2 (8.0%)	0 (0%)	>0.9	3 (9.7%)	4 (29%)	0.2
Bone marrow plasmacytosis	40 (20, 65)	65 (58, 78)	0.053	60 (28, 80)	30 (15, 40)	0.072
ISS at diagnosis			0.3			>0.9
1	12 (55%)	1 (50%)		9 (41%)	4 (40%)	
2	2 (9.1%)	1 (50%)		4 (18%)	1 (10%)	
3	8 (36%)	0 (0%)		9 (41%)	5 (50%)	
17p deletion at diagnosis	1 (4.0%)	0 (0%)	>0.9	1 (3.4%)	1 (7.7%)	0.5
% plasma cells in S-phase at diagnosis	0.006 (0.002, 0.016)	0.004 (0.002, 0.005)	0.4	0.007 (0.003, 0.015)	0.004 (0.001, 0.016)	0.6
MSMART high risk category	9 (36%)	1 (25%)	>0.9	3 (10%)	2 (14%)	0.6
R-ISS at diagnosis						0.5
1	5 (36%)	1 (50%)		3 (27%)	1 (25%)	
2	6 (43%)	1 (50%)		4 (36%)	3 (75%)	
3	3 (21%)	0 (0%)		4 (36%)	0 (0%)	
Concurrent plasma cell leukemia	0 (0%)	0 (0%)		1 (3.6%)	0 (0%)	>0.9
Initial treatment regiment						
Proteasome inhibitor	9 (43%)	1 (33%)	>0.9	10 (43%)	5 (42%)	>0.9
Immunomodulator	15 (71%)	2 (67%)	>0.9	15 (65%)	8 (67%)	>0.9
ASCT performed	12 (60%)	1 (33%)	0.6	8 (38%)	5 (45%)	0.7

*AA* African American, *ASCT* autologous stem cell transplantation, *CH* clonal hematopoiesis, *NHW* non-Hispanic White, *MM* multiple myeloma.

^a^Median (IQR); *n* (%).

^b^*p* values were assessed between Black/AA patients with and without CH and NHW patients with and without CH. Wilcoxon rank sum exact test; Fisher’s exact test were used.

While genotoxic stress and selective pressure on hematopoietic stem cells with an increased fitness is a well-recognized risk factor for CH [3], the increased frequency of CH does not appear to be restricted to patients with previous exposure to cytotoxic therapy [4, 5]. In our cohort, which included untreated patients, similar frequencies of CH were obtained in MM (23%) and in various PCNs (17% of MGUS, 25% of SMM and 22% with amyloidosis). A contribution of treatment-independent factors such as common underlying environmental exposures predisposing to CH and MM, MM-modulated immune dysfunction and alterations in the medullary microenvironment have also been posited [4]. Of distinct interest, an African ancestry-specific germline variant at the locus of an enhancer regulating *TET2* expression was associated with an increased risk of CHIP [6]. We thus sought to evaluate whether the frequency of CHIP may be differentially affected by race among patients with PCNs, yet we found similar frequencies of CH occurrence among Black/AA and NHW patients with PCN. Our findings are in accordance with and expand on previous studies offering more modest representation of Black/AA individuals, affording the largest assessment of CH frequency in this population across different PCNs [1, 7–9].

Previous reports have revealed variable prognostic implications of CH in MM [1, 7]. In our general cohort, while only a trend for an adverse prognostic effect of CH at a VAF ≥ 0.01 was observed, a statistically significant deleterious impact on PFS and OS was documented with a VAF ≥ 0.02. PFS was not differentially altered by self-reported race and ethnicity; however, similarly to the recent study by Peres et al. [9], an adverse effect on OS was noted in the setting of CH for Black/AA patients.

In summary, our study suggests that the prevalence of CH in the setting of PCNs does not significantly differ between Black/AA and NHW individuals. While African-specific germline variants predisposing to CH have been uncovered, our findings support that aging and potential factors related to PCN biology prevailingly influence CH frequency. Among Black/AA patients, our results suggest that CH may however be associated with deleterious implications on OS.

## Supplementary information

Supplemental data

## Data Availability

The NGS data that support this study have been deposited in the National Center for Biotechnology Information (NCBI)’s Sequence Read Archive with BioProject ID PRJNA856503 and can be accessed at http://www.ncbi.nlm.nih.gov/bioproject/856503.
