# Supplementary material for: Distribution of clonal hematopoiesis of indeterminate potential (CHIP) is not associated with race in patients with plasma cell neoplasms
Source: Blood Cancer J. 2022 Jul 26;12(7):112. doi: 10.1038/s41408-022-00706-5 (PMC9325693; doi:10.1038/s41408-022-00706-5)
Supplement: Supplementary file 1 — Supplemental data [file 41408_2022_706_MOESM1_ESM.docx]

**Supplemental Methods and Results**

**Sequencing, bioinformatics pipeline and variant curation**

Libraries were sequenced, in 2x150bp paired mode, to an average of 20 million read pairs per sample on an Illumina HiSeq 4000 allowing an average sequencing depth of ~4,000x with reliable detection of variants with a variant allele frequency (VAF) as low as 0.3%. Raw reads were mapped to the human reference (hg38) with BWA-mem at default settings(1). Duplicates were marked using the Picard MarkDuplicates tool (https://broadinstitute.github.io/picard/). Variants were called with GATK Mutect2(2) using default settings and annotated according to the clinical sequencing nomenclature implemented in Clinical Annotation of Variants (CAVA), a fast and lightweight variant annotation tool(3). We focused on variants predicted to have functional effects, including nonsense, frameshift, stop gain/loss, missense and alteration of splice sites(4, 5). The retained variants were then annotated with Biological Annotation Data Repository (BioR)(6) and subjected to additional filters as below. Common variants were eliminated if they were present in any of the germline variant databases, including 1000 Genomes Project, ExAC, gnomAD and TOPMed, with a minor allele frequency (MAF) >0.5%. To minimize sequencing and mapping artifacts, variants were filtered out if they had a read mapping quality <20, total read depth <30x, alternate read depth <10x, VAF <1%; or if located in the five types of complex genomic regions described in (4) but not in the Catalogue of Somatic Mutations in Cancer (v92). Finally, variants of significant interest were visually inspected over reads alignment using Integrative Genomics Viewer (IGV)(7) and cross-validated with previous studies in MM populations(5, 8). The frequency of detection of CH and size of clonal populations were not correlated with plasmocytic bone marrow infiltration.

**Statistical methods**

Pearson Chi-squared tests and Fisher’s exact tests were used to assess for between-group differences in categorical variables and Wilcoxon rank sum test for continuous variables with non-normal distribution. Assumptions for equality of variance were verified. Logistic regression analyses were used to assess the frequency of CHIP adjusting for age and race and ethnicity. Overall survival was defined as time from diagnosis of plasma cell neoplasm to death from any cause, while progression-free survival was defined as time from diagnosis to progression or death. Progression of disease was defined according to IMWG criteria(9). Survival and time-to-event curves were constructed using the Kaplan-Meier method and compared by the log-rank test. Cox proportional-hazards regression models were used for multivariable analysis to determine hazard ratios and associated confidence intervals. Statistical tests were two-sided with a significance level of 0.05. Statistical analyses were performed using R version 4.0.5.

**Self-reported race/ethnicity and calculated ancestry concordance**

Self-reported race and ethnicity were highly concordant with calculated ancestry. Of the patients who self-identified as Black/AA, the median African ancestry was 79% (range 52-92%). Of the patients who self-identified as NHW, the median African ancestry was 0.8% (range <0.1%-8%).

**Supplemental Table 1.** Characteristics and baseline demographics of study cohort based on reported race

| **Characteristic** | **American Indian/Alaska Native/ Pacific Islander patients, n=1*^1^*** | **Asian patients, n=21*^1^*** | **Black/African American patients, n=64*^1^*** | **Hispanic or Latino patients, n=4*^1^*** | **Non-Hispanic White patients, n=81*^1^*** |
| --- | --- | --- | --- | --- | --- |
| Gender (male) | 0 (0%) | 16 (76%) | 34 (53%) | 3 (75%) | 41 (51%) |
| Age | 60 (60, 60) | 63 (54, 69) | 62 (52, 66) | 59 (49, 72) | 68 (59, 75) |
| Diagnosis |  |  |  |  |  |
| Multiple myeloma | 1 (100%) | 11 (52%) | 29 (45%) | 3 (75%) | 45 (56%) |
| MGUS | 0 (0%) | 4 (19%) | 13 (20%) | 0 (0%) | 12 (15%) |
| SMM | 0 (0%) | 0 (0%) | 6 (9.4%) | 1 (25%) | 13 (16%) |
| Amyloidosis | 0 (0%) | 6 (29%) | 13 (20%) | 0 (0%) | 8 (9.9%) |
| POEMS | 0 (0%) | 0 (0%) | 3 (4.7%) | 0 (0%) | 1 (1.2%) |
| Other | 0 (0%) | 0 (0%) | 0 (0%) | 0 (0%) | 2 (2.5%) |
| Primary cytogenetic abnormality |  |  |  |  |  |
| t(11;14) | 0 (0%) | 10 (48%) | 18 (27%) | 17 (27%) | 16 (20%) |
| t(4;14) | 0 (0%) | 1 (4.8%) | 3 (4.5%) | 3 (4.7%) | 5 (6.2%) |
| t(6;.14) | 0 (0%) | 1 (4.8%) | 1 (1.5%) | 1 (1.6%) | 4 (4.9%) |
| MAF translocations | 0 (0%) | 0 (0%) | 14 (21%) | 14 (22%) | 1 (1.2%) |
| Trisomy no IGH | 0 (0%) | 7 (33%) | 23 (36%) | 2 (50%) | 40 (49%) |
| Other IGH | 0 (0%) | 2 (9.5%) | 4 (6.2%) | 1 (25%) | 10 (12%) |
| Bone marrow plasmacytosis | 90 (90, 90) | 10 (5, 50) | 15 (5, 40) | 35 (28, 50) | 20 (10, 50) |
| Concurrent amyloidosis | 0 (NA%) | 9 (64%) | 15 (43%) | 0 (0%) | 10 (15%) |
| ISS at diagnosis |  |  |  |  |  |
| 1 | 0 (0%) | 3 (30%) | 14 (54%) | 0 (0%) | 14 (42%) |
| 2 | 0 (0%) | 4 (40%) | 4 (15%) | 1 (50%) | 5 (15%) |
| 3 | 1 (100%) | 3 (30%) | 8 (31%) | 1 (50%) | 14 (42%) |
| MSMART High Risk Category | 0 (0%) | 2 (9.5%) | 17 (27%) | 0 (0%) | 9 (11%) |
| R-ISS at diagnosis |  |  |  |  |  |
| 1 | 0 (NA%) | 1 (11%) | 6 (35%) | 0 (0%) | 4 (25%) |
| 2 | 0 (NA%) | 5 (56%) | 8 (47%) | 2 (100%) | 8 (50%) |
| 3 | 0 (NA%) | 3 (33%) | 3 (18%) | 0 (0%) | 4 (25%) |
| Paraprotein subtype |  |  |  |  |  |
| IgG | 0 (0%) | 11 (55%) | 39 (63%) | 2 (50%) | 46 (57%) |
| IgA | 0 (0%) | 2 (10%) | 14 (23%) | 1 (25%) | 21 (26%) |
| LCO | 1 (100%) | 7 (35%) | 9 (15%) | 1 (25%) | 11 (14%) |
| Other | 0 (0%) | 0 (0%) | 0 (0%) | 0 (0%) | 2 (2.5%) |
| Kappa light chain | 1 (100%) | 12 (57%) | 35 (55%) | 2 (50%) | 45 (56%) |
| CH (AF threshold 0.01) | 0 (0%) | 2 (9.5%) | 10 (15%) | 9 (14%) | 24 (30%) |
| DNMT3A | 0 (0%) | 1 (4.8%) | 7 (11%) | 7 (11%) | 12 (15%) |
| TET21 | 0 (0%) | 1 (4.8%) | 7 (11%) | 6 (9.4%) | 13 (16%) |
| ASXL1 | 0 (0%) | 0 (0%) | 2 (3.0%) | 2 (3.1%) | 3 (3.7%) |
| Maximal VAF | NA (NA, NA) | 0.31 (0.18, 0.45) | 0.03 (0.02, 0.05) | 0.02 (0.02, 0.04) | 0.06 (0.02, 0.15) |
| VAF (additive) | NA (NA, NA) | NA (NA, NA) | 0.00 (0.00, 0.00) | 0.04 (0.03, 0.05) | 0.27 (0.20, 0.48) |
| VAF (multiplicative) | NA (NA, NA) | NA (NA, NA) | 0.04 (0.03, 0.05) | 0.00 (0.00, 0.00) | 0.01 (0.01, 0.01) |
| CH (AF threshold 0.02) | 0 (0%) | 0 (0%) | 3 (4.5%) | 3 (4.7%) | 10 (12%) |
| TET2 | 0 (0%) | 0 (0%) | 0 (0%) | 0 (0%) | 8 (9.9%) |
| DNMT3A | 0 (0%) | 0 (0%) | 2 (3.0%) | 2 (3.1%) | 2 (2.5%) |
| ASXL12 | 0 (0%) | 0 (0%) | 1 (1.5%) | 1 (1.6%) | 1 (1.2%) |
| Any treatment received | 1 (100%) | 15 (71%) | 40 (49%) | 21 (47%) | 28 (56%) |
| Initial treatment regimen |  |  |  |  |  |
| Proteasome inhibitor-based | 1 (100%) | 8 (42%) | 12 (22%) | 0 (0%) | 18 (26%) |
| Immunomodulator-based | 0 (0%) | 4 (21%) | 20 (36%) | 2 (50%) | 26 (38%) |
| Best Response to Initial Treatment |  |  |  |  |  |
| Stringent complete response | 0 (NA%) | 1 (6.7%) | 1 (3.1%) | 0 (0%) | 0 (0%) |
| Complete response | 0 (NA%) | 3 (20%) | 4 (12%) | 1 (33%) | 7 (18%) |
| Very good partial response | 0 (NA%) | 6 (40%) | 7 (22%) | 1 (33%) | 17 (41%) |
| Partial response | 0 (NA%) | 3 (20%) | 15 (47%) | 0 (0%) | 8 (20%) |
| Minimal response | 0 (NA%) | 1 (6.7%) | 1 (3.1%) | 0 (0%) | 0 (0%) |
| Stable disease | 0 (NA%) | 1 (6.7%) | 4 (12%) | 1 (33%) | 8 (20%) |
| ASCT Received | 0 (0%) | 8 (38%) | 17 (32%) | 1 (25%) | 16 (25%) |
| Progression of disease | 0 (NA%) | 10 (56%) | 21 (47%) | 3 (100%) | 28 (56%) |

^1^Median (IQR); n (%). ASCT: autologous stem cell transplantation, MGUS: monoclonal gammopathy of undetermined significance. VAF: variant allele frequency

**Supplemental Figures**

A
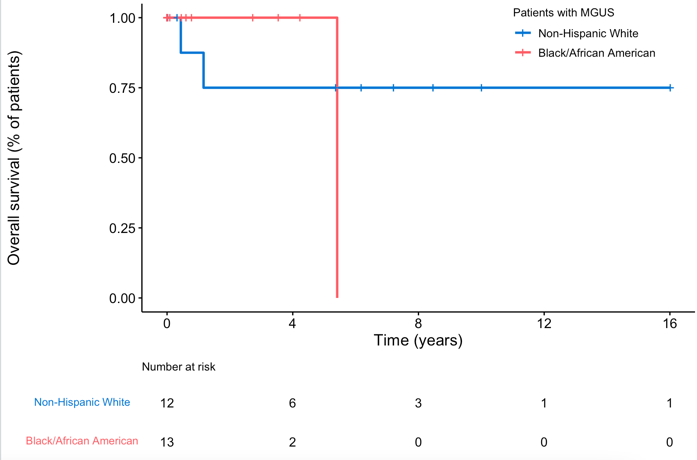
 B
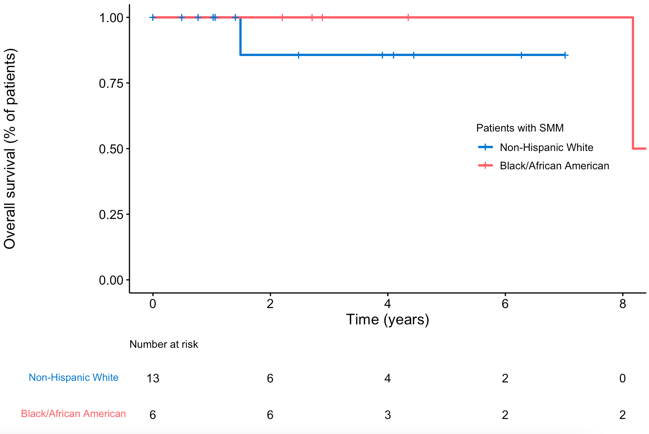
 C
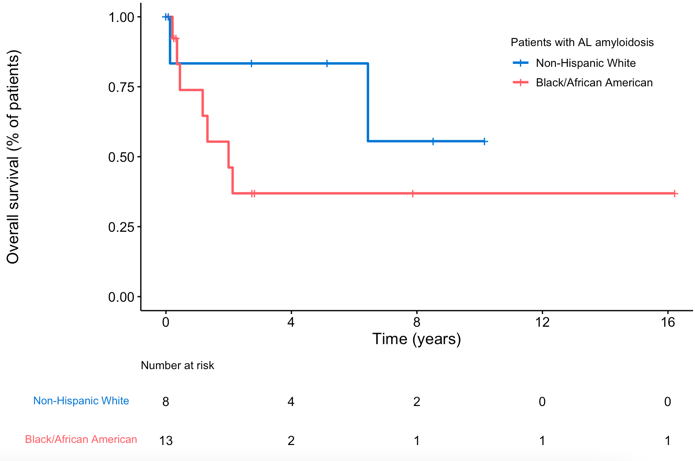


**Supplemental Figure 1.** Overall survival of different plasma cell neoplasms based on race. (A) Overall survival of patients with MGUS (B) Overall survival of patients with smoldering multiple myeloma (C) Overall survival of patients with AL amyloidosis.

A
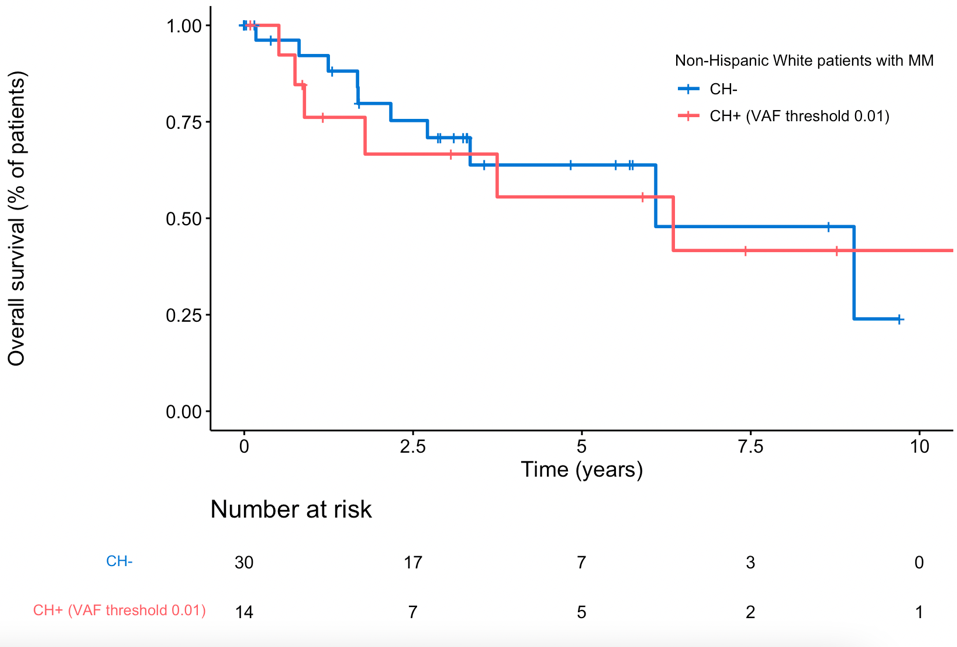
B
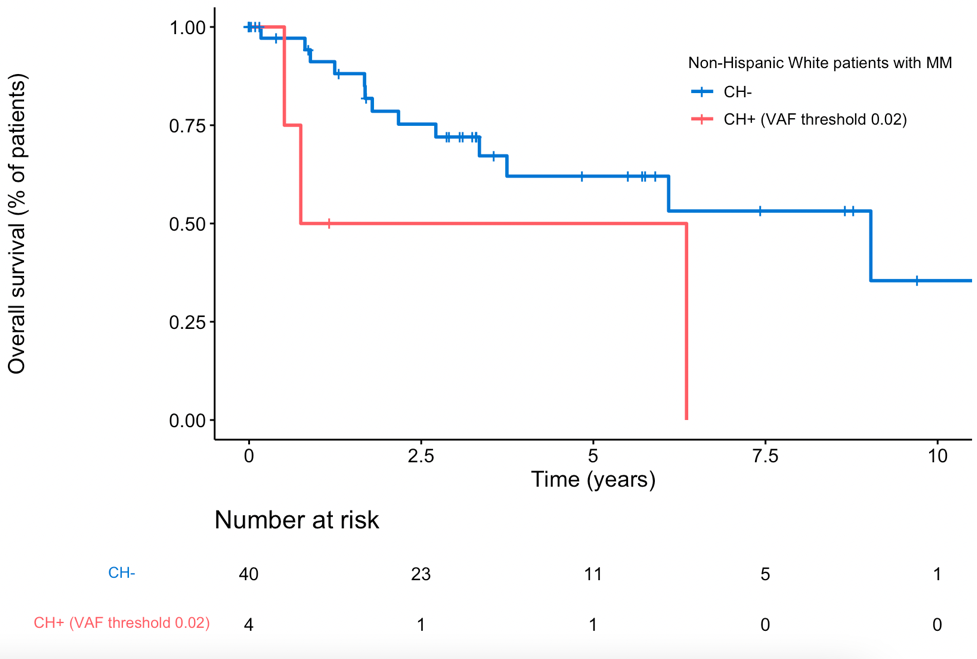


**Supplemental Figure 2.** Overall survival of non-Hispanic White patients with multiple myeloma based on clonal hematopoiesis status at an allele frequency threshold of 0.01 (A) and 0.02 (B) *One patient had no available follow-up data.*

A
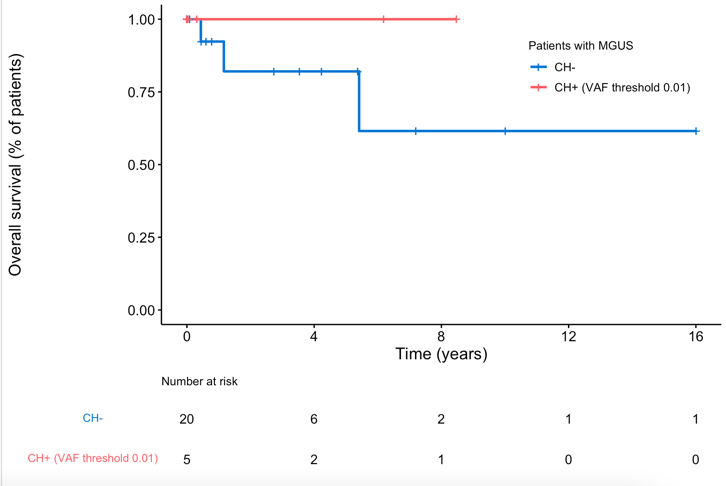
B
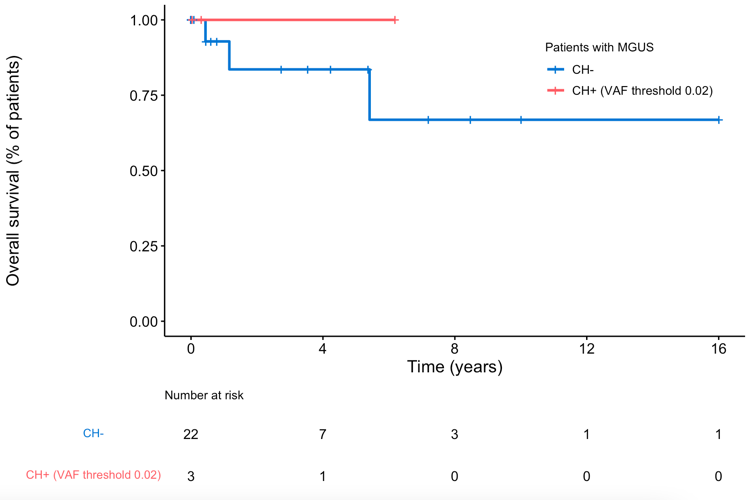


C
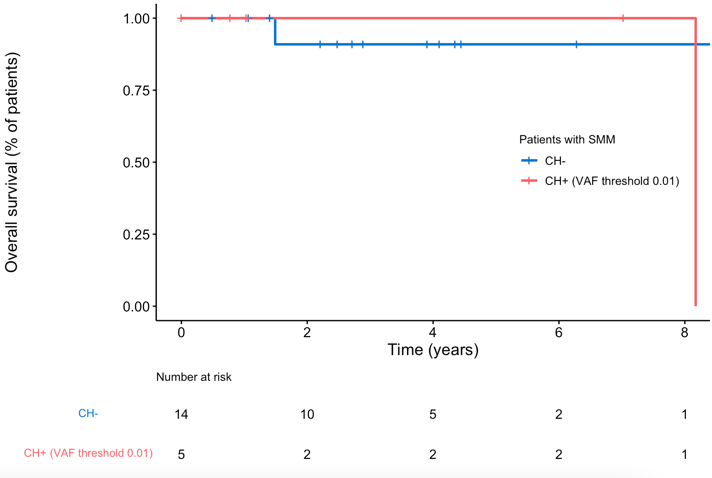
 D
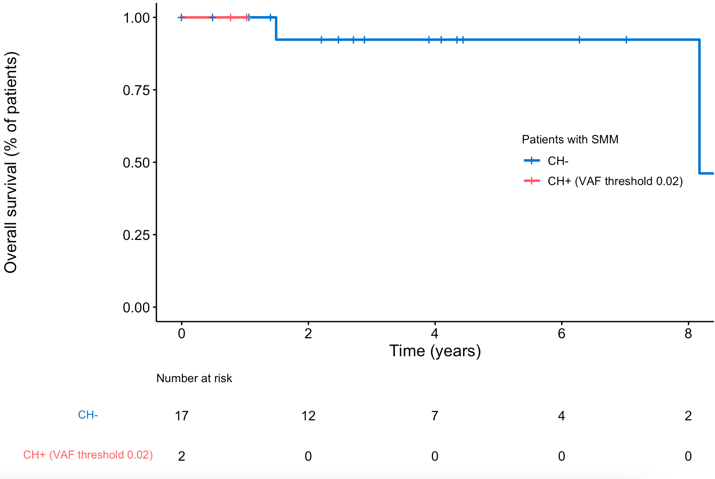


E
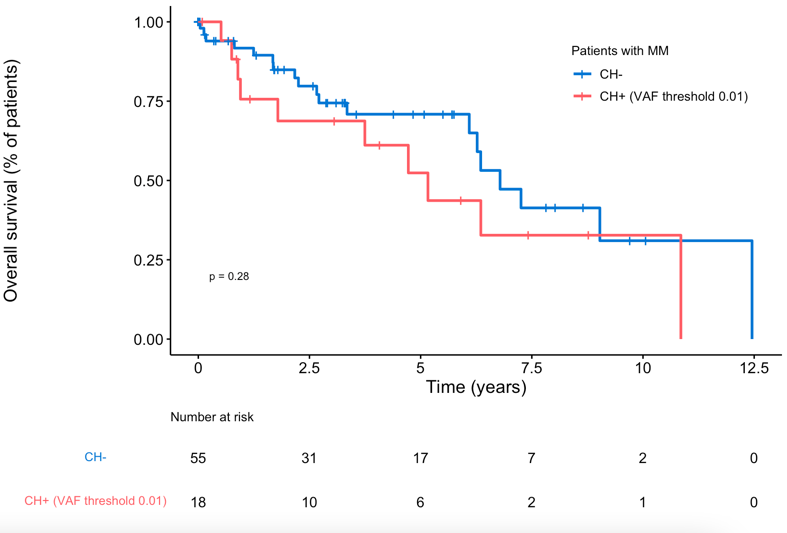
 F
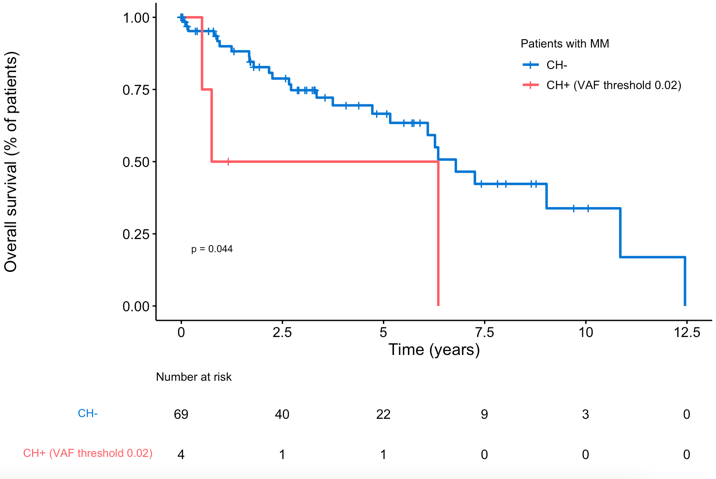


G
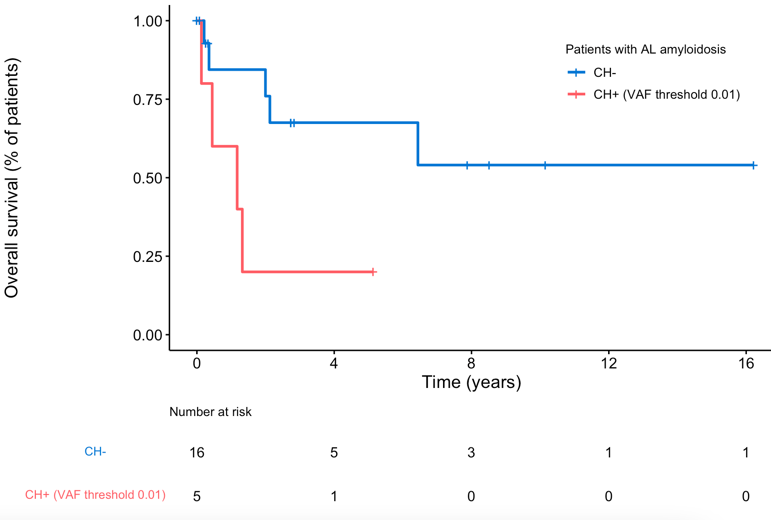
H
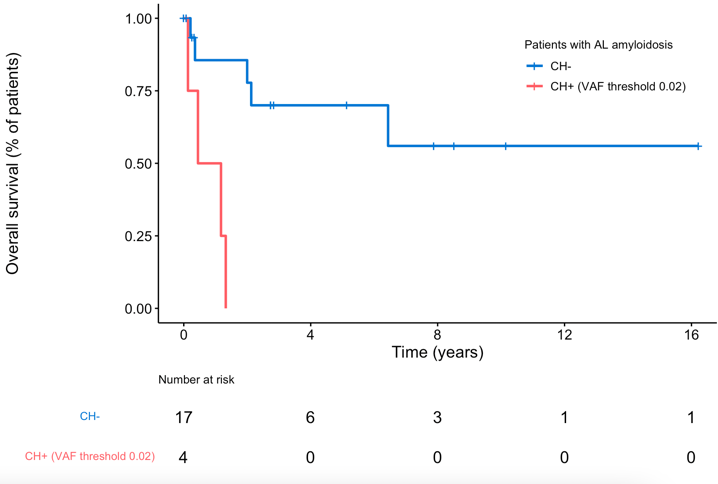


**Supplemental Figure 3.** Overall survival of different plasma cell neoplasms based on clonal hematopoiesis (CH). Overall survival of patients with MGUS based on CH status as defined by a variant allele frequency threshold of 0.01 (A) and 0.02 (B). Overall survival of patients with smoldering multiple myeloma (SMM) based on CH status as defined by a variant allele frequency threshold of 0.01 (C) and 0.02 (D). Overall survival of patients with multiple myeloma (MM) based on CH status as defined by a variant allele frequency threshold of 0.01 (E) and 0.02 (F). Overall survival of patients with AL amyloidosis based on CH status as defined by a variant allele frequency threshold of 0.01 (G) and 0.02 (H)


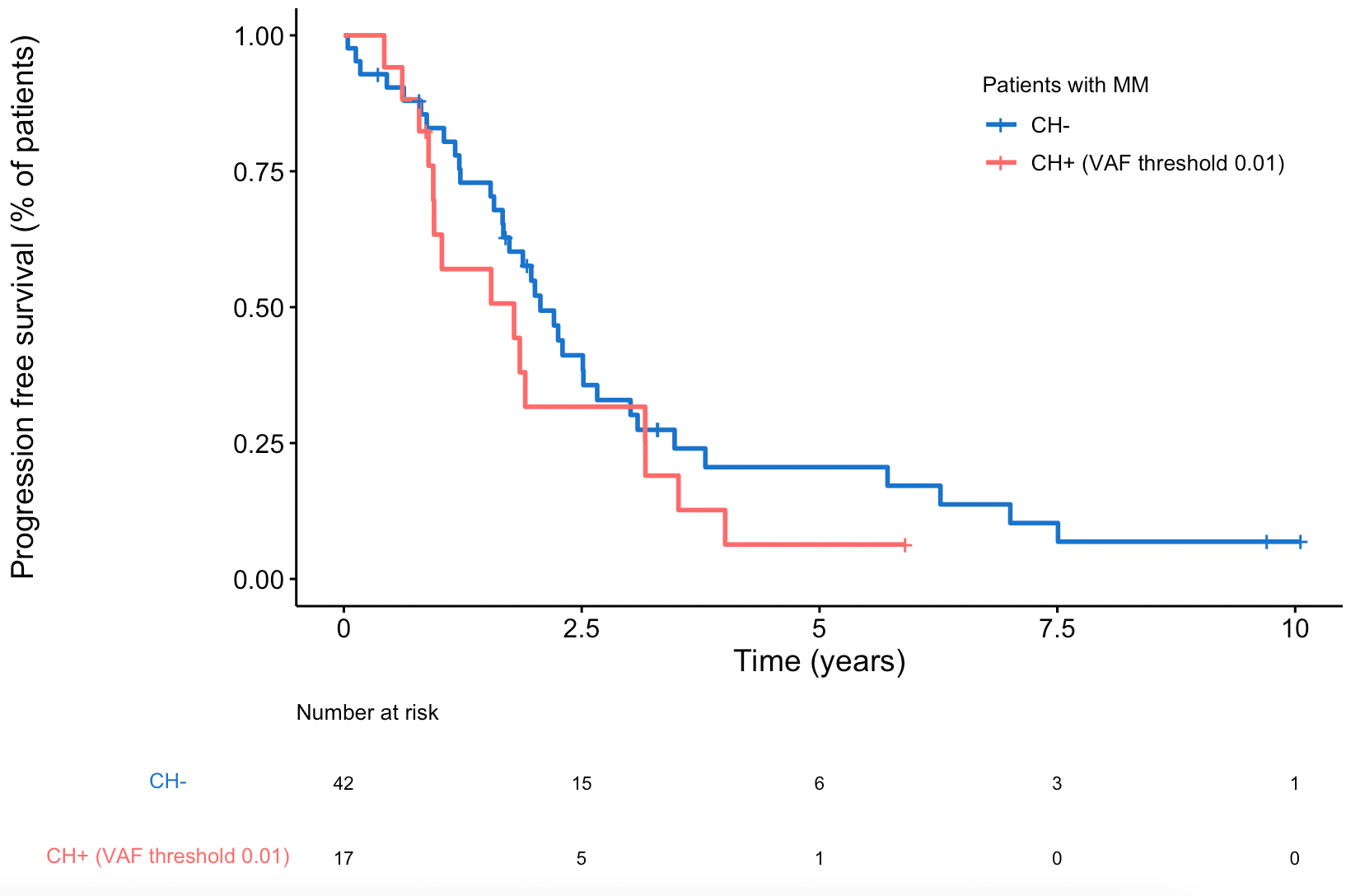


**Supplemental Figure 4.** Progression-free survival of patients with multiple myeloma based on clonal hematopoiesis status (allele frequency threshold 0.01). *Fifteen patients had no available progression data and were excluded from analyses.*


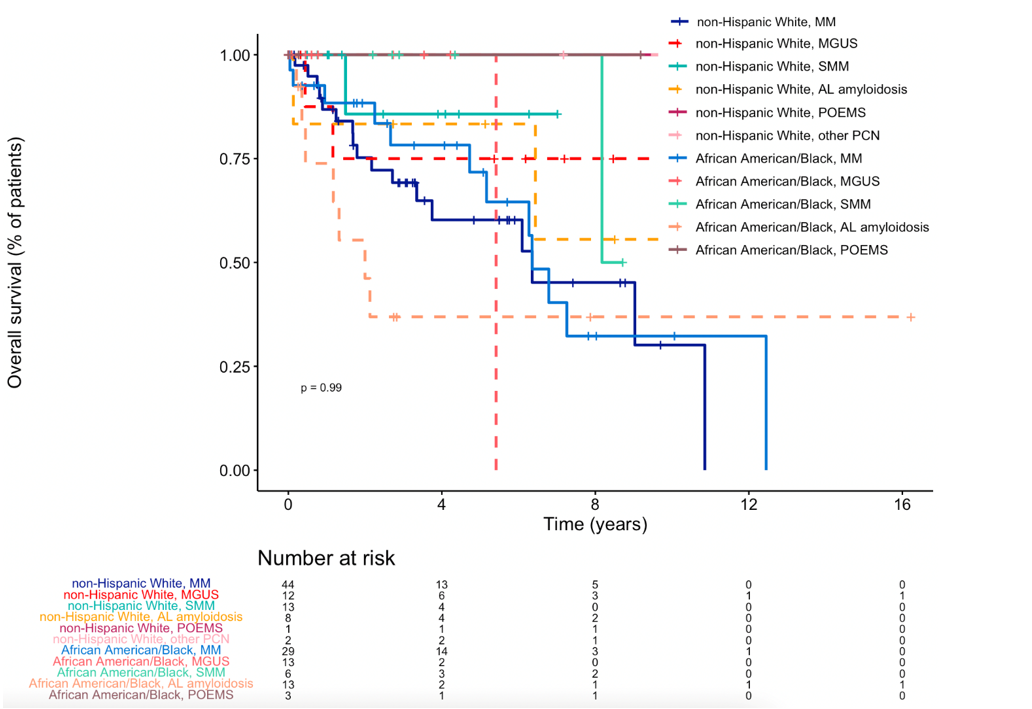


**Supplemental Figure 5.** Overall survival of entire cohort based on race and ethnicity and with stratification on PCN type (HR=1. 05, 95% CI: 0.53 – 1.86; p=0.99)


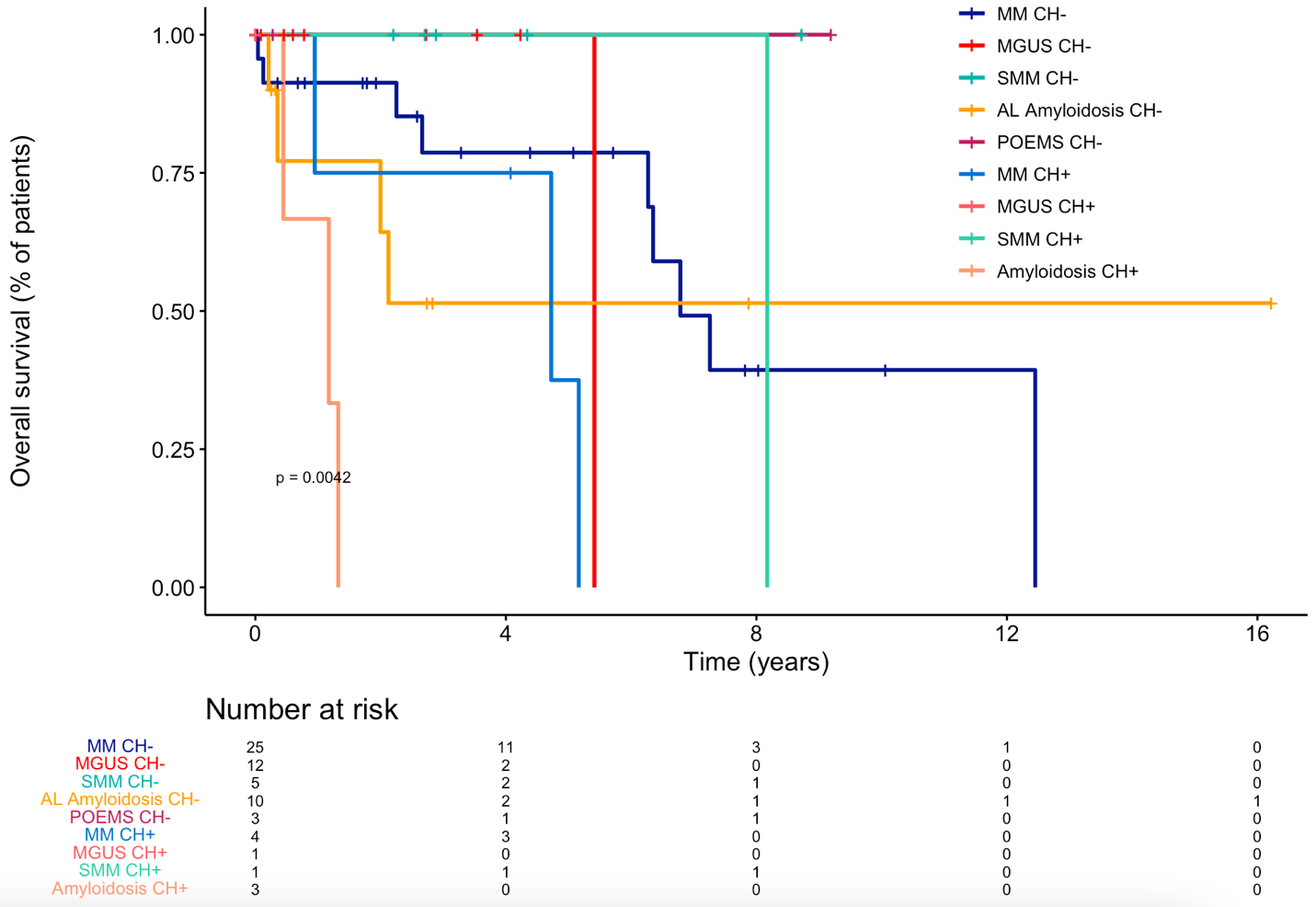


**Supplemental Figure 6.** Overall survival of Black/African American patients according to CH status (VAF ≥0.01) and stratified on plasma cell neoplasm type (HR=4.57, 95% CI: 1.48 – 14.1; p=0.008. CH: clonal hematopoiesis, PCN : plasma cell neoplasm.


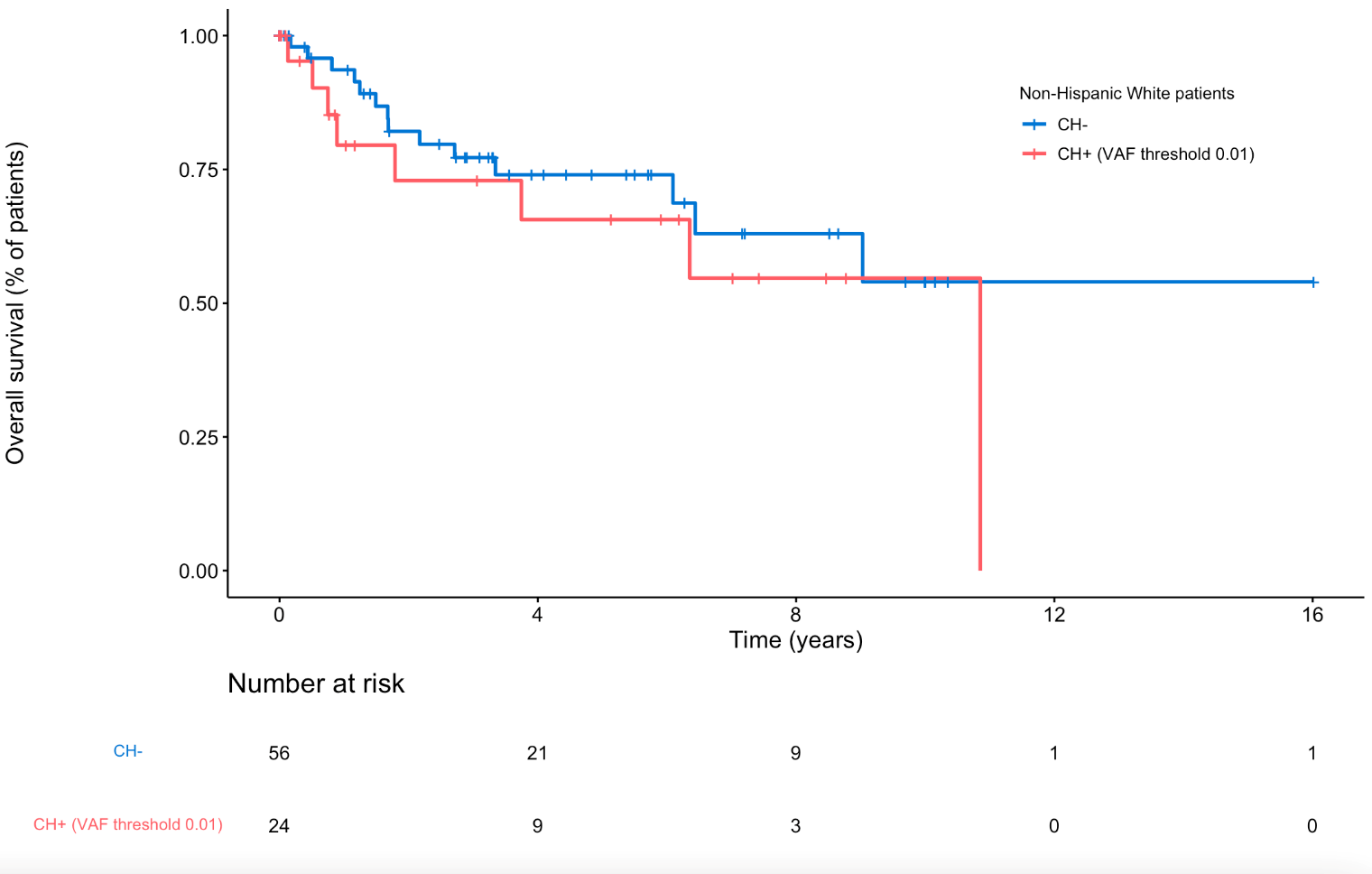


**Supplemental Figure 7.** Overall survival of Non-Hispanic White patients with a plasma cell neoplasm according to CH status (VAF ≥0.01). CH: clonal hematopoiesis.

**References**

1. Li H, Durbin R. Fast and accurate short read alignment with Burrows-Wheeler transform. Bioinformatics. 2009;25(14):1754-60.

2. Cibulskis K, Lawrence MS, Carter SL, Sivachenko A, Jaffe D, Sougnez C, et al. Sensitive detection of somatic point mutations in impure and heterogeneous cancer samples. Nat Biotechnol. 2013;31(3):213-9.

3. Munz M, Ruark E, Renwick A, Ramsay E, Clarke M, Mahamdallie S, et al. CSN and CAVA: variant annotation tools for rapid, robust next-generation sequencing analysis in the clinical setting. Genome Med. 2015;7:76.

4. Genovese G, Kahler AK, Handsaker RE, Lindberg J, Rose SA, Bakhoum SF, et al. Clonal hematopoiesis and blood-cancer risk inferred from blood DNA sequence. N Engl J Med. 2014;371(26):2477-87.

5. Jaiswal S, Fontanillas P, Flannick J, Manning A, Grauman PV, Mar BG, et al. Age-related clonal hematopoiesis associated with adverse outcomes. N Engl J Med. 2014;371(26):2488-98.

6. Kocher JP, Quest DJ, Duffy P, Meiners MA, Moore RM, Rider D, et al. The Biological Reference Repository (BioR): a rapid and flexible system for genomics annotation. Bioinformatics. 2014;30(13):1920-2.

7. Robinson JT, Thorvaldsdottir H, Wenger AM, Zehir A, Mesirov JP. Variant Review with the Integrative Genomics Viewer. Cancer Res. 2017;77(21):e31-e4.

8. Tahri S, Mouhieddine TH, Redd RA, Lampe LM, Nilsson KI, El-Khoury H, et al. Clonal hematopoiesis is associated with increased risk of progression of asymptomatic Waldenstrom macroglobulinemia. Blood Adv. 2021.

9. Rajkumar SV, Dimopoulos MA, Palumbo A, Blade J, Merlini G, Mateos MV, et al. International Myeloma Working Group updated criteria for the diagnosis of multiple myeloma. Lancet Oncol. 2014;15(12):e538-48.
